# Functional temozolomide sensitivity testing of patient-specific glioblastoma stem cell cultures is predictive of clinical outcome

**DOI:** 10.1016/j.tranon.2022.101535

**Published:** 2022-09-15

**Authors:** Erlend Skaga, Evgeny Kulesskiy, Swapnil Potdar, Ioannis Panagopoulos, Francesca Micci, Iver A. Langmoen, Cecilie J. Sandberg, Einar O. Vik-Mo

**Affiliations:** a Vilhelm Magnus Lab, Institute for Surgical Research and Department of Neurosurgery, Oslo University Hospital, P.O. Box 4950 Nydalen, 0424, Oslo, Norway; b Institute for Molecular Medicine Finland, FIMM, University of Helsinki, Tukholmankatu 8, 00290, Helsinki, Finland; c Section for Cancer Cytogenetics, Institute for Cancer Genetics and Informatics, The Norwegian Radium Hospital, Oslo University Hospital, Montebello, P.O. Box 4954 Nydalen, 0424, Oslo, Norway; d Institute of Clinical Medicine, Faculty of Medicine, University of Oslo, P.O. Box 1112 Blindern, 0317, Oslo, Norway

**Keywords:** Glioblastoma, Glioblastoma stem cells, Temozolomide, Drug sensitivity, Tumor heterogeneity, GSC, Glioblastoma stem cell, TMZ, Temozolomide, GBM, Glioblastoma, DSS, Drug sensitivity score, MGMT, O6-methylguanine-DNA methyltransferase, DSRT, Drug sensitivity and resistance testing

## Abstract

Serum-free culturing of patient-derived glioblastoma biopsies enrich for glioblastoma stem cells (GSCs) and is recognized as a disease-relevant model system in glioblastoma (GBM). We hypothesized that the temozolomide (TMZ) drug sensitivity of patient-derived GSC cultures correlates to clinical sensitivity patterns and has clinical predictive value in a cohort of GBM patients. To this aim, we established 51 individual GSC cultures from surgical biopsies from both treatment-naïve primary and pretreated recurrent GBM patients. The cultures were evaluated for sensitivity to TMZ over a dosing range achievable in normal clinical practice. Drug efficacy was quantified by the drug sensitivity score. MGMT-methylation status was investigated by pyrosequencing. Correlative, contingency, and survival analyses were performed for associations between experimental and clinical data. We found a heterogeneous response to temozolomide in the GSC culture cohort. There were significant differences in the sensitivity to TMZ between the newly diagnosed and the TMZ-treated recurrent disease (p <0.01). There was a moderate correlation between MGMT-status and sensitivity to TMZ (r=0.459, p=0.0009). The relationship between MGMT status and TMZ efficacy was statistically significant on multivariate analyses (p=0.0051). We found a predictive value of TMZ sensitivity in individual GSC cultures to patient survival (p=0.0089). We conclude that GSC-enriched cultures hold clinical and translational relevance by their ability to reflect the clinical heterogeneity in TMZ-sensitivity, substantiate the association between TMZ-sensitivity and MGMT-promotor methylation status and appear to have a stronger predictive value than MGMT-promotor methylation on clinical responses to TMZ.

## Highlights

• TMZ sensitivity of GSCs is correlated to their MGMT methylation status.
• TMZ sensitivity of GSCs *ex vivo* is predictive of improved patient survival.
• Individualized GSC cultures can be utilized for functional precision medicine.

## Introduction

In glioblastoma (GBM), a highly heterogeneous and malignant brain tumor, the development of serum-free cell culturing techniques of patient-derived biopsies has proven to be a robust preclinical model for the human disease compared to conventional culturing techniques using serum [1,2]. The serum-free technique enriches the cell culture for the subpopulation of glioblastoma stem cells (GSCs) [3]. The GSCs possess important qualities as a model system in GBM that includes the ability to preserve key genetic alterations of the parent tumor [4,5], to maintain a range of individual clones from the same tumor [6,7], and preserve the invasive phenotype *in vivo* [8,9]. Furthermore, patient-derived GSC cultures have clinical predictive relevance, as tumorsphere formation of GSCs *in vitro* and a GSC gene signature both are independent negative predictors of patient survival [10,11].

Despite retaining pivotal qualities as a preclinical model of GBM, the clinical translational value of the GSCs has yet to be established. A recent study reported on the clinical predictive value of *ex vivo* temozolomide (TMZ) sensitivity in GSCs [12]. They quantified drug efficacy using IC_50_-values, which are described to capture limited information about drug efficacy when tested over a dose range [13] They, moreover, evaluated efficacy of TMZ in drug concentrations that far exceeds what can be considered clinically achievable. The use of such supra-therapeutic drug concentrations is discouraged in preclinical testing [14]. The recently developed drug sensitivity score (DSS) is an improved quantification of drug efficacy that captures and integrates the multiparametric dose-response relationship of drug efficacy into a single metric named the drug sensitivity score (DSS) [13]. Compared to traditional methods of drug efficacy evaluations, the DSS has been shown to better distinguish active from inactive drugs when evaluating efficacy over a dose range [13]. Clinical utility of drug sensitivity testing using the DSS has recently been demonstrated in different tumor entities. For instance, drug sensitivity testing of patient-derived sarcoma cells *ex vivo* correlates to clinical responses [15], and drug sensitivity testing of patient-derived leukemic cells can uncover patient-specific drug sensitivities that induce clinical remissions in chemorefractory disease [16]. We therefore sought to explore whether drug sensitivity to temozolomide in patient-derived GSCs using the DSS hold predictive value when compared to clinical outcome in a heterogeneous cohort of both treatment-naïve and recurrent GBM.

## Material and methods

### Brain tumor biopsies and cell cultures

Glioblastoma biopsies were obtained from 51 informed and consenting patients undergoing surgery for GBM (primary GBM n=40, recurrent GBM n=11) at Oslo University Hospital, Norway. The study was approved by The Norwegian Regional Committee for Medical Research Ethics (REK 07321b and 2017/167). Histopathological diagnostics was performed according to the WHO classification. Cell cultures were established both from the tumor biopsy and ultrasonic aspirate generated during surgery and cultured under tumorsphere forming conditions in serum-free, growth factor enriched media, as previously described [3]. Both the tumor biopsies and the ultrasonic aspirate were immediately after the surgery transferred to the laboratory. The tumor tissues were subsequently dissociated mechanically with scalpels. The ultrasonic aspirate was centrifuged to remove debris and erythrocytes. Cells were then dissociated using trypsin-EDTA (Invitrogen) for 5 min at 37°C before filtered to a single cell suspension and cultured in low-attachment flasks in EGF and bFGF (both R&D Systems) supplemented cell media consisting of DMEM/F12 (Invitrogen), HEPES (Lonza), B27-supplement (Invitrogen) and penicillin/streptomycin (Lonza). Cells were incubated at a density of 10^5^ cells/mL. Once weekly, 50% of the cell culture media was replaced, while EGF and bFGF were added to the medium twice weekly. When spheres reached a size where the core of the spheres turned dark (around 100 µm) or when cells that solely proliferated with adherent morphology covered the bottom of the flask, the cultures were enzymatically dissociated into singe cells and re-cultured.

### Cell viability assay

Pilot experiments with an established sensitive (GBM4) and a resistant (GBM11) GSC culture were performed to establish adequate cell numbers per well and incubation time after exposure to TMZ (Supplementary Fig. S1). Cells were plated at 2500 cells/well in a 96-well plate (Sarstedt) under sphere forming conditions. After 24 h, vehicle (0.5% DMSO), TMZ and positive control (1.25 µM sepantronium bromide, SelleckChem) were added, and the cells further incubated for 10 days. Media or drug was not replenished during the incubation period. TMZ concentrations covered a 5-point dose-escalating pattern (0.4 – 250 µM, each concentration tested in 5 biological replicates) capturing the area of clinical relative drug concentrations in cerebrospinal fluid and plasma [17]. Cell viability was assessed using the Cell Proliferation Kit II XTT (Roche) solution with incubation for 24 h before absorbance was analyzed on a PerkinElmer EnVision.

### Sphere-forming assay

Cells were plated at 500 cells/well in a 96-well plate (Sarstedt) under sphere-forming conditions. After 24 h, TMZ, negative (0.5% DMSO) and positive (1.25 µM sepantronium bromide) control were added, and the cells further incubated for 10 days. TMZ concentrations covered a 5-point dose-escalating pattern (0.4 – 250 µM, each concentration tested in 5 biological replicates). After 10 days, the spheres were stained using Thiazolyl Blue Tetrazolium Bromide (Sigma-Aldrich) 4 h prior to image acquisition and counted using an automated colony counter (GelCount). Spheres >30 µm in diameter were included in the analysis and results reported relative to negative control.

### Generation of dose-response curves and scoring

For drug sensitivity and resistance testing (DSRT), raw data was corrected for background absorbance and scaled with reference to the positive (sepantronium bromide) and negative (DMSO) control to generate relative viability percent inhibition. The resulting set of values was then fitted with non-linear least squares method. The half-maximal inhibitory concentration (IC_50_), slope, and maximum and minimum response from the curve fitting were further used to calculate the Drug Sensitivity Score (DSS) and the resulting DSRT was analyzed using the Breeze application, as previously described [13,18].

### MGMT-promoter methylation

Genomic DNA was isolated from cells using the Maxwell 16 Cell DNA Purification Kit and the Maxwell 16 Instrument (Promega) before treatment with the EpiTect Bisulfite Kit (Qiagen). qPCR was performed using the MGMT Pyro Kit (Qiagen) according to the manufacturer's instructions. The samples were then processed in the PyroMark Q24 system (Qiagen), and the obtained data were analyzed with the PyroMark CpG Software (Qiagen). MGMT promoter methylation was calculated as an average of the rate of methylation of CpG-sites 76-79. Cultures with an average methylation of ≥9% were considered MGMT methylated according to previously published and clinically evaluated validation [19].

### Statistical considerations

Data analysis and graphic presentation were undertaken using GraphPad Prism 9.0, Keynote 11.1, Microsoft Excel 16.5, and R. Population characteristics are presented by descriptive statistics. Differences between groups were compared using an unpaired two-sided t-test. The correlation analyses were performed using Pearson correlation. Contingency analyses were performed using chi-squared statistics with Fisher's exact test. Survival analyses were calculated from the time of surgery to the time of death by the Kaplan–Meier method. Regression analyses were performed by Cox regression modeling. A p-value <0.05 was considered significant.

## Results

In this cohort, 51 individual GSC cultures were established from patients undergoing surgery for either a newly diagnosed (n=40) or recurrent (n=11) GBM. We, and others, have previously described the ability of the GSC culture system to preserve patient-specific traits and the malignant phenotype in cultures proliferating both as spheres and with adherent morphology [20,21]. Under serum-free and growth factor enriched conditions, 45 (88%) formed free-floating tumorspheres while 6 (12%) proliferated with adherent morphology. Of the 51 GSC cultures, 21 (41%) have previously been in depth characterized for stem cell properties of which 19/21 (90%) formed invasive tumors upon xenografting to immunodeficient mice within 15 weeks [21], [22], [23], [24]. The current experiments were performed at a median of passage seven – safely within the timeframe for which we have previously demonstrated that cultures preserve their patient specific characteristics [3]. Further baseline characteristics of the patients and cultures are provided in Table 1.

**Table 1:** Patient and cell culture characteristics. Baseline patient and cell culture characteristics. ^#^9/11 patients received standard RT/TMZ ad modum Stupp before surgery for recurrent GBM. One patient received RT only due to advanced age and one patient received RT only due to a primary diagnosis of anaplastic astrocytoma.

	Primary GBM	Recurrent GBM
No. of patients	40	11
Age, median (range)	64 (46-84)	54 (35-79)
Male	28 (74%)	9 (82%)
IDH-status		
wild-type	30 (75%)	11 (100%)
mutated	1 (3%)	-
unknown	9 (22%)	-
MGMT status (parent tumor)		
methylated	11 (28%)	2 (18%)
unmethylated	16 (40%)	7 (64%)
unknown	13 (32%)	2 (18%)
Resection grade		
total	8 (20%)	3 (27%)
subtotal	29 (72%)	8 (73%)
not recorded	3 (8%)	-
Oncological treatment		
RT/TMZ	35 (88%)	9^#^
RT only	2 (7%)	2^#^
unknown	3 (5%)	-
Overall survival from diagnosis, months	11.1	15.4
Culture morphology		
spheroids	36 (90%)	9 (82%)
adherent	4 (10%)	2 (18%)
Culture passage experiments, median (range)	7 (3-19)	7 (3-9)

### Patterns of sensitivity to temozolomide in glioblastoma stem cells

**Fig. 1:**
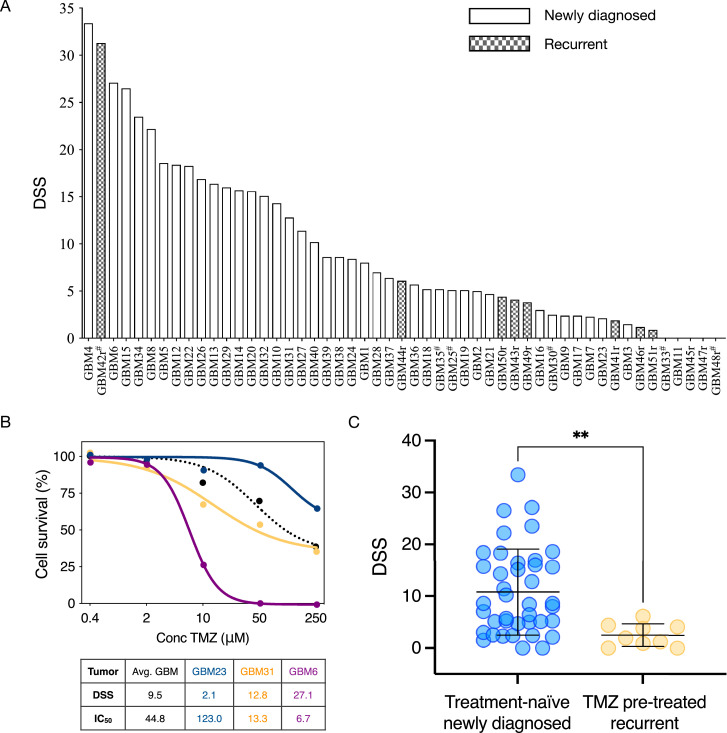
Efficacy of temozolomide in a heterogeneous population of GSC cultures. A) TMZ efficacy across the entire cell culture cohort presented as absolute effects by the DSS. B) Dose-response curves of sensitivity to TMZ in three cell cultures (colored lines) along with the average dose-response curve (dotted line) from the entire cohort to demonstrate a minimal (blue), moderate (orange) and high (purple) sensitivity to TMZ. IC_50_-values are in µM concentrations. C) Distribution of sensitivity to TMZ between the GSC cultures derived from treatment-naïve GBM (n=40) along with the sensitivity to TMZ in the GSC cultures derived from recurrent GBM that had received TMZ during the previous disease course (n=9). ** p<0.01.

We next evaluated the individual cultures sensitivity to TMZ over a dose range covering previously defined, clinically relevant concentrations [17]. Like the heterogeneous response to TMZ seen in clinical practice [25], the sensitivity of the individual GSC cultures to TMZ varied considerably from resistant (DSS = 0) to highly sensitive (maximum DSS = 50, where higher DSS equals increased sensitivity, Fig. 1A). The average DSS to TMZ across the culture cohort was 9.5. From a large-scale evaluation of drug sensitivity and resistance patterns in GSCs, we have previously defined a DSS ≥ 10 as a threshold for at least moderate drug efficacy (Fig. 1B) [22]. Using this threshold, 19 cultures (37%) were defined with a moderate to high sensitivity to TMZ, of which only one culture was derived from a recurrent GBM. Of notice, this patient had previously not been treated with TMZ before recurrence due to advanced age. 10/11 of the cultures derived from recurrent disease were classified as resistant to TMZ. Thus, the patterns of sensitivity to TMZ were more heterogeneous among cultures derived from the treatment-naïve patients. Comparing the TMZ sensitivity in the cultures derived from treatment-naïve patients (n= 40) to cultures derived from patients with recurrence of GBM that previously had received TMZ (n = 9) revealed significant differences in TMZ sensitivity (p<0.01, Fig. 1C). Sphere-formation is an independent negative predictor of survival in GBM patients [10]. We found, however, no significant differences in the sensitivity to TMZ at the group level between the different cell cultures morphologies (average DSS sphere cultures 9.8 (n=45) vs. average DSS adherent cultures 7.4 (n=6), p=0.52, data not shown).

We next assessed whether TMZ sensitivity affects the sphere-forming capacity in cultures categorized as either highly sensitive (DSS >15) or resistant (DSS <5). We compared five highly sensitive cultures (GBM4, GBM5, GBM12, GBM15 and GBM22) to five TMZ-resistant cultures (GBM3, GBM11, GBM23, GBM46r and GBM47r) using additional sphere-forming assays. Automated quantification of sphere count, total area of spheres and average diameter of the spheres, demonstrated that although cultures were categorized as highly sensitive to TMZ the main effect seemed to be related to the size of the spheres rather than the total number (Supplementary Fig. S1 and S2). This indicates that TMZ, even at high concentration and in highly sensitive GSC cultures, do not eradicate all spheres in this assay (Supplementary Fig. S1 and S2).

### Conformity of MGMT status between parent tumor and corresponding GSC culture

**Fig. 2:**
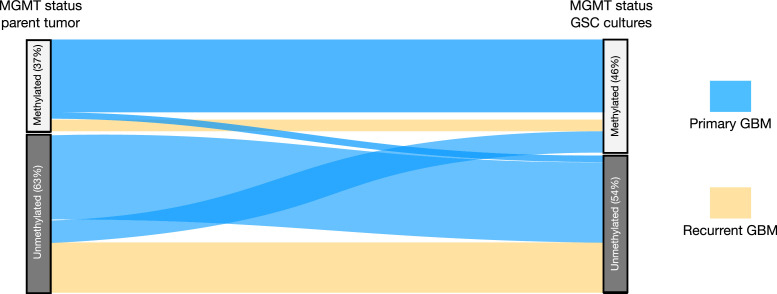
Conformity in MGMT status. Sankey plot of MGMT methylation status between the parent tumor and the corresponding GSC cultures (n=35 pairs) demonstrate conformity in 30 out of 35 pairs.

Clinical response to TMZ treatment in GBM patients is correlated to the methylation status of the MGMT promoter [25]. We used a clinically verified methylation status of 9% as a cut-off to dichotomize the cultures as either methylated (≥9%) or unmethylated (<9%) [19]. MGMT methylation status from the parental tumor was available for 36 (71%) of GSC cultures, in whom nine were from recurrent tumors. Among the cell cultures, two had (GBM24, GBM35) technical errors during MGMT methylation quantification and was excluded from the analysis. This led to a total of 35 pairs where we had methylation status of both the parent tumor and the corresponding GSC culture. The conformity of the MGMT methylation status between the tumor and the corresponding cell culture was high (86%, Fig. 2). Five cultures, all from primary GBM, exhibited a disparity in methylation status category upon culturing. One culture changed from an unmethylated tumor to a methylated cell culture, while four cultures changed to unmethylated cell cultures from methylated tumors (Fig. 2).

### Temozolomide sensitivity and correlation to MGMT promoter methylation status

**Fig. 3:**
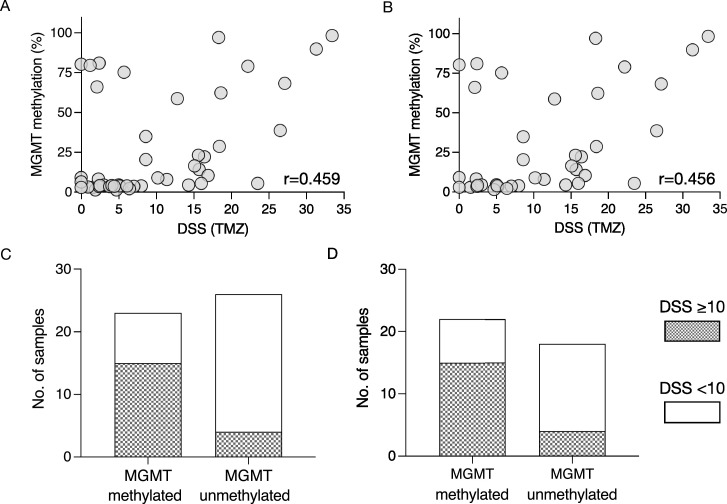
Associations between TMZ sensitivity and MGMT methylation status in GSCs. A) Scatter plot of sensitivity to TMZ (absolute effect) and the grade of methylation of the MGMT promoter in the entire cohort of GSCs (n=49). B) Scatter plot of sensitivity to TMZ (absolute effect) and the grade of methylation of the MGMT promoter in GSCs derived from treatment naïve GBMs (n=39). Contingency analyses of all C) and TMZ-naïve D) cultures on the associations between sensitivity to TMZ (DSS) and MGMT methylation status.

We further compared the MGMT methylation status of the individual GSC cultures to the cultures’ respective sensitivity to TMZ. Among the cell cultures were 23/49 (47%) methylated. Of the TMZ-naïve cultures 22/40 (55%) were methylated. Using all cultures in whom results from both TMZ sensitivity and MGMT methylation status were present (n=49), we found a moderate, statistically significant correlation between the variables (r=0.459, p=0.0009, Fig. 3A). Selecting only the cultures derived form TMZ-naïve patients (n=40) similarly revealed a moderate, statistically significant correlation (r=0.456, p=0.0031) between TMZ sensitivity and MGMT methylation status (Fig. 3B). There was a small cluster of cultures that were insensitive to TMZ despite having a high percentage of methylation in the MGMT promoter. On the contrary, there were also a small cluster of cultures with a moderate to high sensitivity to TMZ that were MGMT unmethylated (Fig. 3A-B). This points to MGMT methylation status to partly explain patterns of TMZ sensitivity in GBM. Contingency analyses of all (Fig. 3C) and the TMZ-naïve (Fig. 3D) cultures, further established the connection between MGMT promoter methylation and sensitivity to TMZ (p=0.0005 and p=0.0051, respectively).

### Temozolomide sensitivity ex vivo is associated with improved patient survival

**Fig. 4:**
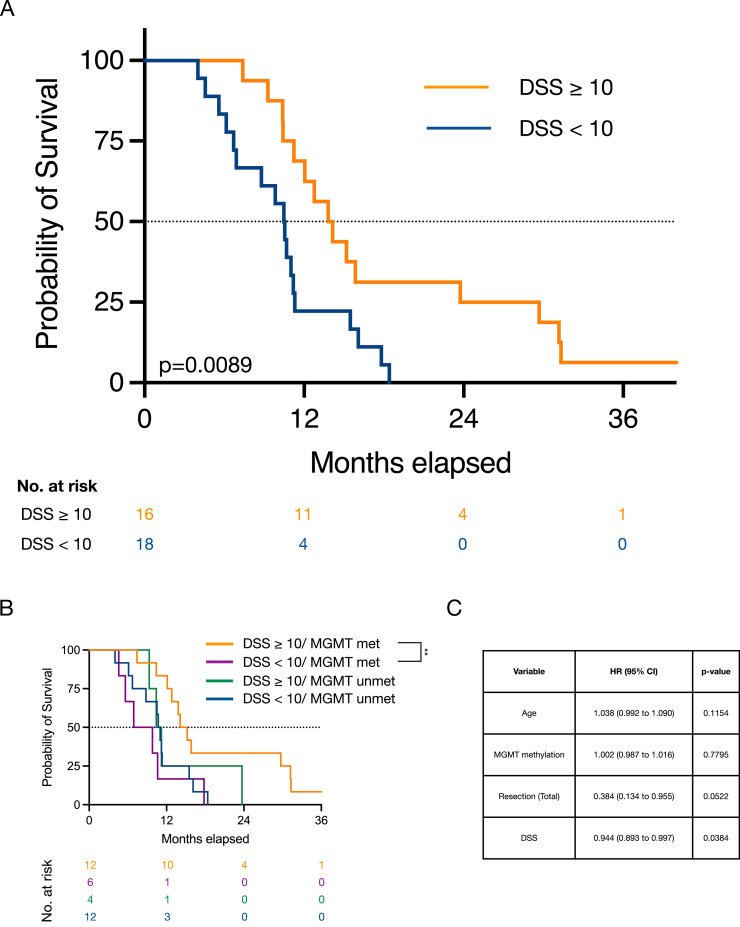
Clinical predictive value of TMZ sensitivity ex vivo related to patient survival. A) The classification of the GSC cultures from treatment-naïve GBMs into sensitive (DSS ≥ 10) or resistant (DSS <10) demonstrated a statistically significant improved survival in the cultures classified as TMZ-sensitive. B) Subclassification of the cultures according to both DSS and MGMT demonstrate the predictive power to of the DSS in the methylated group. C) Cox proportional regression analysis with hazard ratios (HR) of the known variables to influence survival (age, MGMT status, resection grade) compared to DSS.

A preclinical model has limited clinical translational value if the results are not applicable to a clinical setting. We therefore investigated the clinical predictive value of DSRT of GSCs *ex vivo* using the DSS. First, we reviewed the medical records of the newly diagnosed patients that received TMZ treatment during the concomitant (w/radiotherapy) and/or the adjuvant treatment phase. Of the 40 patients with a newly diagnosed GBM, 35 (88%) received TMZ-treatment, two patients (5%) received only RT due to advanced age, while the oncological treatment could not be established in three patients (7%). Second, we compared the *ex vivo* TMZ sensitivity data of individual GSC cultures to the survival of patient in the group of GBM patients that had received TMZ. We missed survival data in one patient, such as the total number of patients for the survival analyses were 34. Using our pre-defined cut-off of DSS ≥ 10 as threshold for TMZ sensitivity in the GSC cultures, we found by the Kaplan-Meier method a survival benefit in the patients categorized as sensitive to TMZ (median survival 14.0 vs. 10.5 months, p=0.00189, Fig. 4A). This effect was not apparent using a lower threshold level of DSS ≥ 5 to dichotomize the sensitive (DSS ≥ 5) to the insensitive (DSS < 5, p=0.2478, Supplementary Fig. S3A). The DSS is developed to capture the effect of a drug over a dose-range compared to the commonly used point estimates such as IC_50_. There was, however, a strong correlation between DSS and a lower IC_50_ in the individual cultures (r=-0.9336, p=<0.0001, Supplementary Fig. S3B). The relationship followed an exponential decay curve (r=0.92) such as solely relying in IC_50_ to establish TMZ efficacy may not identify GSCs with robust dose-response relationships (Supplementary Fig. S3C). A recent publication described a survival benefit in the patients whom their GSCs had an IC_50_ of TMZ <180 μM [12], a concentration of TMZ that exceeds the concentrations possible to reach in patients [17]. We further explored the predictive value of similar point estimates of drug efficacy in our GSC culture cohort. We were not able to select for patients with a survival benefit using a cut-off value of 50 μM which is commonly used as a C_max_ level of TMZ in plasma (Supplementary Fig. S3D). A survival benefit was, however, found lowering the level of cut-off to 20 μM (Supplementary Fig. S3E), confirming the correlative relationship between the IC_50_-estimate and DSS.

The MGMT promoter methylation is widely accepted as a favorable predictive marker for clinical response to TMZ [25]. We did not, however, find a statistically significant survival benefit in the patients with a MGMT methylated GSC culture compared to the unmethylated (median survival 13.3 vs. 10.6 months, p=0.1187, Supplementary Fig. S4A). Stratifying the cultures according to DSS and MGMT-status and further comparing the results to patient survival confirmed, however, the predictive power of *ex vivo* TMZ sensitivity testing in the MGMT methylated patients (p=0.0151, Fig. 4B). This effect was not apparent in the group of MGMT unmethylated patients (p=0.4655). However, this latter analysis may be limited due to few cultures in the group of TMZ sensitive and MGMT unmethylated tumors (n=4).

To analyze the predictive power of DSS on patient survival we additionally performed Cox regression that included the additional variables of patient age and extent of resection (EOR) at primary surgery (total vs. subtotal resection) that are known to be independent factors that influence survival [26]. Using log-rank test, we found a statistically significant survival benefit in the cohort of patients that underwent total resection of their tumor (median survival 17.2 vs. 11.1 months, p=0.0208). On the other hand, using the threshold of 70 years commonly employed in clinical trials in GBM [27], we found no statistically significant survival benefit in the cohort of patients ≤ 70 years (median survival 11.7 months vs. 10.7 months, p=0.0777). Although the limited number of patients for the regression analysis warrants cautious interpretation, using patient age, MGMT and DSS and continuous variables and EOR as a dichotomized variable, we found that the DSS was the only statistically significant variable (p=0.0384, Fig. 4C).

## Discussion

This study confirms and further establish the predictive value of patient derived GSCs in terms of correlating *ex vivo* drug sensitivity to clinical data. Our main results are the experimental confirmation of a heterogeneous drug sensitivity patterns to TMZ in the newly diagnosed disease that are correlated to the methylation status of the MGMT-promoter, the development of TMZ-resistance in the recurrent disease and the clinical value of TMZ-sensitivity in GSCs to predict improved patient survival.

We found that GSCs derived from the newly diagnosed disease were highly heterogeneous in terms of sensitivity to TMZ. This heterogeneity was established at the experimental level from the beginning of the development of the GSC model system [28]. It corresponds well to the heterogeneous response to TMZ in patients [29]. Importantly, we found that the GSCs derived from TMZ-treated recurrent disease, that in principle should be TMZ resistant, were so. This observation has previously been described in smaller cohorts of recurrent GSC cultures [12,30], as such this finding substantiates the ability of individualized GSCs to reflect the clinical reality. It also provides experimental data to the clinical finding that TMZ rechallenge upon tumor regrowth provides limited survival benefit [31]. TMZ sensitivity is, however, tightly linked to the methylation status of the MGMT promoter. This is firmly established in clinical practice as a routine molecular investigation in clinical pathology [25]. The limited benefit of TMZ at the population level for MGMT promotor unmethylated GBM has led to discussion whether it should be withheld in this subgroup of patients – to potentially provide experimental therapies earlier in the disease course [32]. Molecular mechanisms involved in resistance to TMZ are, however, more complex than solely the methylation status of the MGMT promoter [33]. At the individual level there are patients classified with a methylated MGMT promoter with limited treatment effects of TMZ, while on the other hand, there are patients classified with an unmethylated MGMT promoter that significantly benefit from TMZ [25]. In accordance, we found a moderate, statistically significant correlation between TMZ DSS in the individual culture and their corresponding MGMT status. Our contingency analyses, however, confirmed the association at the group level. As the predictive power of MGMT methylation in clinical care is firmly established, the lack of correlative survival benefit in the GSC cultures categorized as methylated is in our study likely related to the limited number of patients in the analysis.

We found a relationship between TMZ-sensitivity in the individual GSC cultures and patient survival. These results add to the biological, clinical, and translational value of individualized GSC cultures as a simplified, however, predictive model of the parent tumor. Their biological relevance and ability to resemble the human disease has been well documented over the past two decades [8,9,34]. In addition, the GSCs ability to confer increased resistance to current oncological treatment provide a biological rationale and an explanation for the inevitable regrowth of GBM [35,36]. The sphere-forming ability of GSCs has by independent groups been described to be a negative predictor for patient survival – pointing to the clinical relevance of the GSC model [10,12,37]. One group has further substantiated the clinical and translational value of the GSCs by demonstrating a clinical value of GSC cultures as TMZ sensitivity is predictive of patient survival [12]. However, as they rightly discuss, their experimental setup has shortcomings with only a few cultures categorized as TMZ-sensitive and drug efficacy evaluations using point estimates in concentrations that exceeds clinically relevant concentrations. These limitations warrant independent confirmation. In this study, we provide results that add and extends to their findings by confirming that individualized GSCs hold clinical value in predicting patient survival evaluated by TMZ sensitivity along with establishing a threshold level of DSS to categorize a culture as TMZ-sensitive. Our study further found that compared to the established predictive variables of patient survival (age, EOR, MGMT methylation), the DSS was the only variable reaching statistical significance. We emphasize, however, that this result should be interpreted with caution and is likely due to the limited number of patients that could be included in the survival analyses.

A preclinical model system is always limited to the real-world situation in patients. In the case of patient derived GSC cultures, selective culturing enriches for a defined cell population. Although GSCs represent a well-characterized population with clinically relevant features, this selection results in loss of parts of the cellular spectrum found in the parental tumor. A promising emerging model platform that may provide better preservation of the clonal heterogeneity and an improved host-to tumor interaction are GBM organoids [38]. Despite some biological advantages, GBM organoid models have not been validated for their clinical predictive ability in terms of correlative analyses to proliferative capacity, invasiveness, and prediction of treatment responses to the same extent as individualized GSC cultures. For both model systems, however, the true translational value remains uncertain as there are very few prospective investigations to whether patient derived individualized GSC cultures or organoids can guide useful treatment decision.

Although the clinical effectiveness is established, the absolute effects of TMZ treatment in a GBM population are limited [29]. The complex heterogeneous biology of GBMs suggests that new treatments should be more patient tailored. Individualized GSC cultures represent a model system that could facilitate that. A retrospective study recently reported on the clinical correlates of a few GBM cases between *in vitro* drug sensitivity and clinical responses using serum-free cell culturing of patient-derived biopsies, thus providing indirect evidence of potential clinical utility for individualized treatments [39]. Two independent groups have explored both the predictive value and clinical utility of *in vitro* chemosensitivity testing of patient-derived biopsies in high-grade gliomas [40], [41], [42]. One group found that TMZ-sensitivity was predictive of delayed recurrence in IDH-mutant high-grade gliomas [40]. They further described both the clinical utility of *ex vivo* chemosensitivity assays to guide patient treatment and a survival benefit when treated patients were compared to historical data [41]. An independent group reported similarly on the predictive value and clinical utility for treatment decisions using patient-derived biopsies in a cohort of high-grade gliomas [42]. Although encouraging for the field of functional precision medicine, the reports are of limited translational value in the era of GSC-directed therapy as they used serum when culturing the biopsies known to impact the biological resemblance to the disease [1,8]. Thus, whether individualized GSC-cultures can provide guidance for useful treatment decision awaits prospective investigations.

Precision medicine in cancer has traditionally been linked to genomics for identification of druggable targets and subsequently match the genomic alteration to a targeted drug [43]. More functional approaches (i.e. functional precision medicine) are, however, based on direct exposure of cancer cells to various anticancer drugs to identify the most effective option [44]. To increase the likelihood of success for a functional precision medicine platform in oncology, the results of drug sensitivity testing *ex vivo* must be predictive of clinical outcome. The data must, moreover, be generated in a turnaround time that allow for clinical translation. Our data implies that individualized cell cultures in GBM can be utilized to identify patient-specific treatment options. We have previously further described the feasibility to utilize GSC cultures in the recurrent disease for drug sensitivity testing within a turnaround time to permit clinical utility [21]. However, there are several hurdles the field of functional precision medicine must overcome to implement the technology into the clinic [44]. On a broader scale, the most pressing seems to be the lack of standardization. Across different functional precision medicine platforms, the experimental set-up for data generation is far from uniform. This includes the decision on tumor model systems (e.g., spheroids vs. organoids), anticancer drugs and concentrations (e.g., single doses vs. dose-response), methods for quantification of drug effects (e.g., IC_50_ vs. DSS), incubation time and final readout (e.g., viability, apoptosis, cytotoxicity etc.). The present study provides, however, a framework for both model and readout system to promote functional precision medicine in GBM.

In summary, we have substantiated the ability of the GSC model system to provide clinical correlate to TMZ-sensitivity patterns, the development of TMZ-resistance in the recurrent disease and the associations to MGMT-methylation status. We have also confirmed and further established the predictive value of patient derived GSCs in terms of correlating *ex vivo* drug sensitivity to clinical data. Collectively, this supports the translational value of the GSC model to represent a clinical useful model system of its parent tumor with the ability to serve as a reductionistic and living *ex vivo* model system that can be utilized for identification of individualized treatment options and functional precision medicine.

## Funding

This work has been funded by the 10.13039/100008730Norwegian Cancer Society (GRANT#144402) and 10.13039/501100009562Regional Health Authorities (Helse Sør-Øst, GRANT#2021039).

## Data availability statement

The data sets generated and analyzed in this study are available from the corresponding author upon reasonable request.

## CRediT authorship contribution statement

**Erlend Skaga:** Conceptualization, Methodology, Formal analysis, Investigation, Data curation, Writing – original draft, Writing – review & editing, Visualization. **Evgeny Kulesskiy:** Software, Formal analysis, Writing – review & editing. **Swapnil Potdar:** Software, Formal analysis, Writing – review & editing. **Ioannis Panagopoulos:** Investigation, Data curation, Writing – review & editing. **Francesca Micci:** Investigation, Data curation, Writing – review & editing. **Iver A. Langmoen:** Resources, Supervision, Project administration, Writing – review & editing, Funding acquisition. **Cecilie J. Sandberg:** Resources, Supervision, Project administration, Writing – review & editing. **Einar O. Vik-Mo:** Conceptualization, Methodology, Supervision, Project administration, Writing – original draft, Writing – review & editing, Funding acquisition.

## Declaration of Competing Interest

The authors declare that there are no conflicts of interest. The funders had no role in the design of the study, in the collection, analyses or interpretation of data; in the writing of the manuscript, or in the decision to publish the results.

## Appendix Supplementary materials

Image, application 1

Image, application 2
