# Supplementary material for: Functional temozolomide sensitivity testing of patient-specific glioblastoma stem cell cultures is predictive of clinical outcome
Source: Transl Oncol. 2022 Sep 15;26:101535. doi: 10.1016/j.tranon.2022.101535 (PMC9483808; doi:10.1016/j.tranon.2022.101535)
Supplement: Supplementary file 1 [file mmc1.docx]

**Supplementary figure legends**

**Supplementary Figure S1.** Experimental set-up of TMZ sensitivity testing in GSCs. A) – C) Sphere-forming assays of a sensitive (GBM4) and a resistant (GBM11) GSC culture demonstrating the effect of TMZ in higher concentrations in GBM4 after 10 days of incubation by mainly reducing the average sphere diameter and total area of the spheres as compared to the resistant GSC culture. D) and E) Proliferation of the same two GSC cultures (GBM4 and GBM11) exposed to negative (0.5% DMSO) and positive (1.25 µM sepantronium bromide) control at 5-, 7- and 10-days incubation. F) After 10 days of incubation, a clear dose-response pattern emerged in the sensitive GSC culture.

**Supplementary Figure S2.** Sphere-forming capacity in TMZ-sensitive versus TMZ-resistant GSC cultures.

**Supplementary Figure S3.** Clinical predictive ability of *ex vivo* drug sensitivity testing in GSCs. A) Survival of glioblastoma patients analyzed on a lower threshold for sensitivity (DSS ≥5). B) Scatter plot demonstrating the correlation between DSS and IC_50._ C) Dose-response curves demonstrating to cultures with similar IC_50_, but with highly differing DSS. D) Survival of glioblastoma patients analyzed on a threshold of IC_50_>50 μM. E) Survival of glioblastoma patients analyzed on a threshold of IC_50_>20 μM.

**Supplementary Figure S4.** Survival of the treatment-naïve GBM patients stratified according to A) MGMT methylation status of the GSC cultures, B) total versus subtotal resection and C) age > and ≤ 70 years.
