# Supplementary material for: Functional temozolomide sensitivity testing of patient-specific glioblastoma stem cell cultures is predictive of clinical outcome
Source: Transl Oncol. 2022 Sep 15;26:101535. doi: 10.1016/j.tranon.2022.101535 (PMC9483808; doi:10.1016/j.tranon.2022.101535)

Supplementary Figure S1

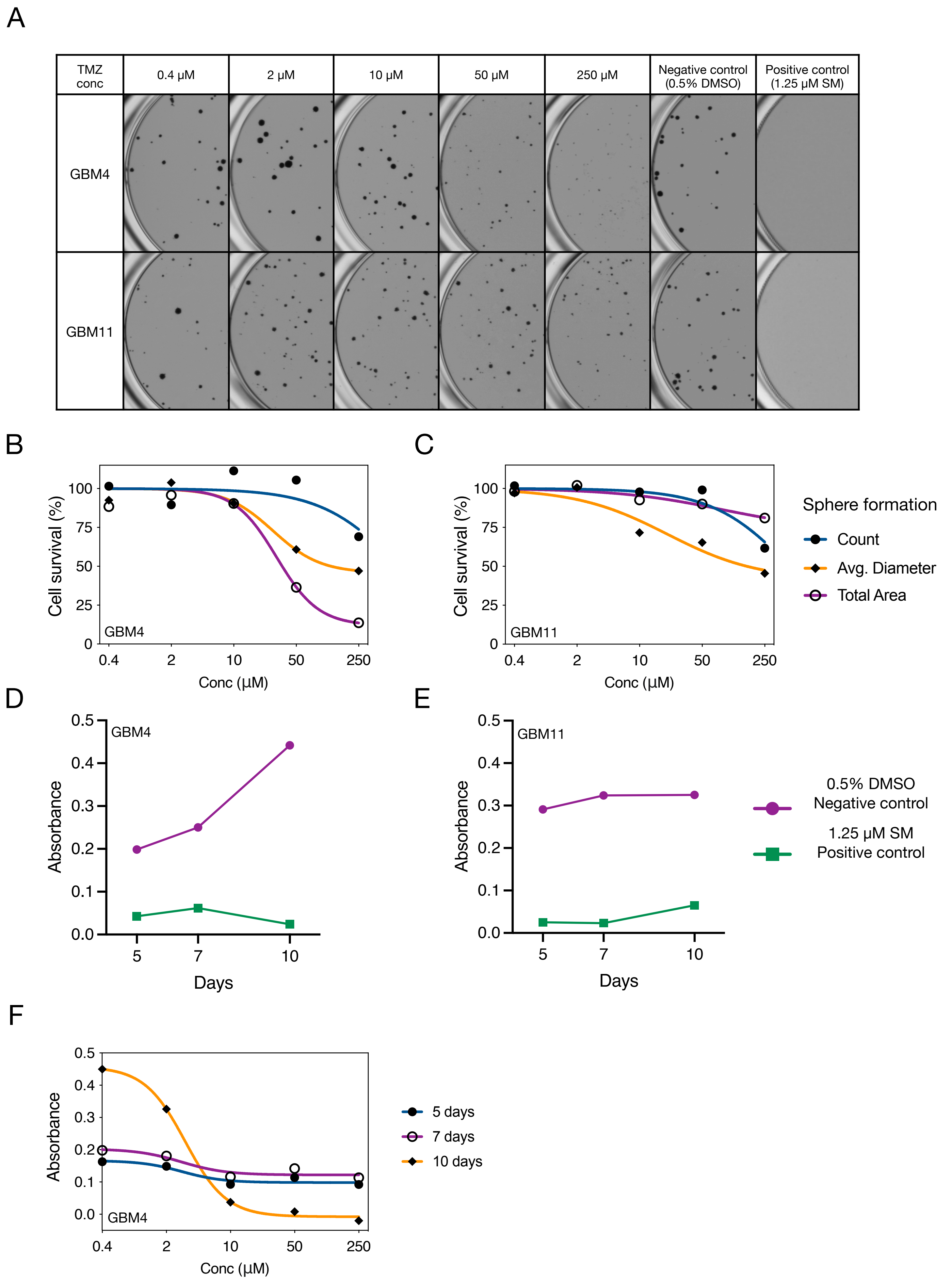

Supplementary Figure S2

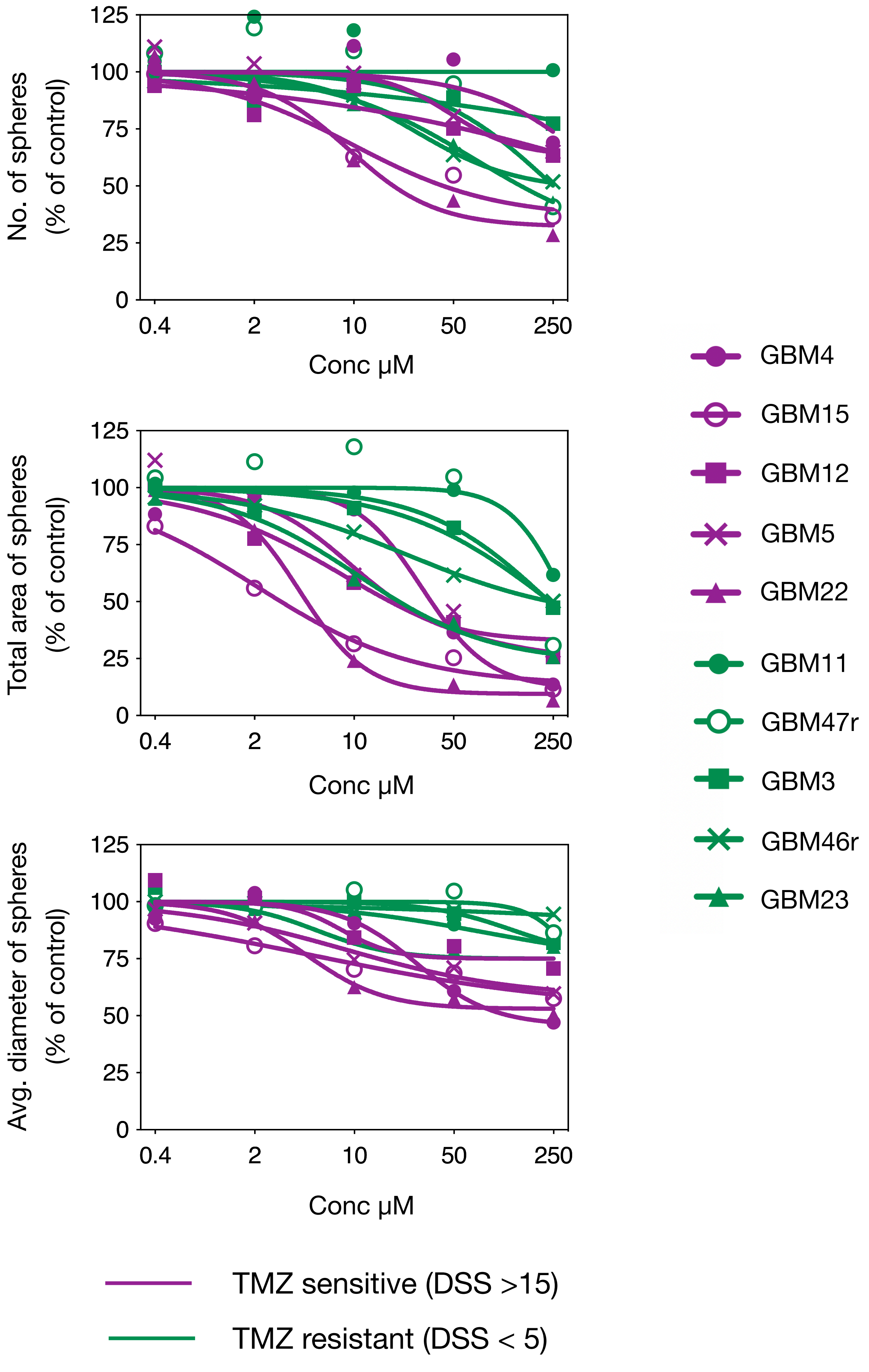

Supplementary Figure S3

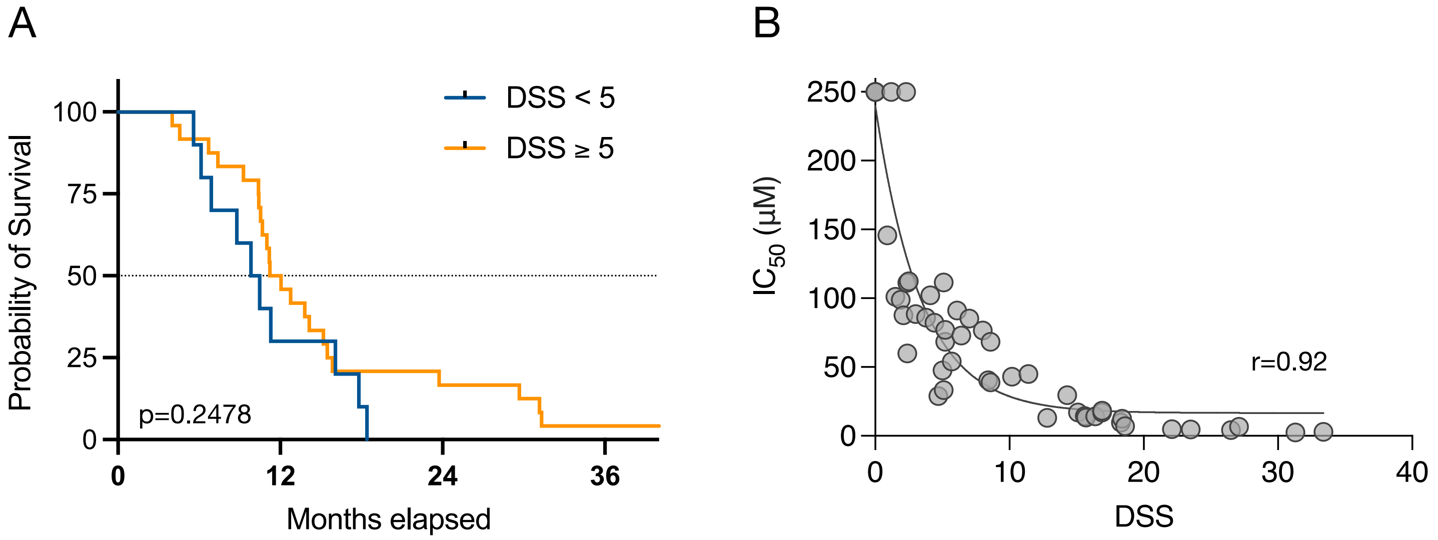

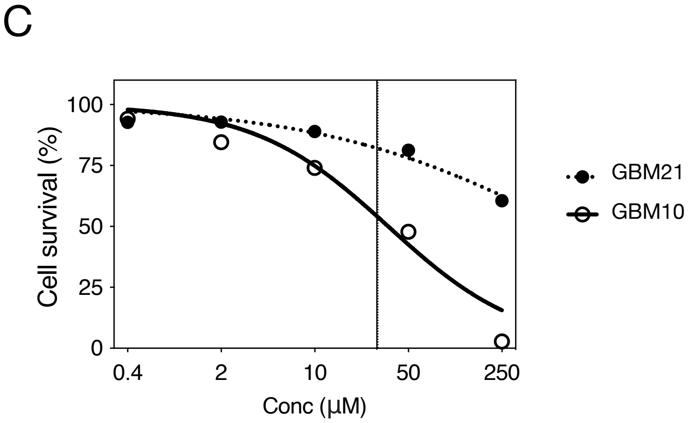

Tumor	GBM21	GBM10
**DSS**	4.7	14.3
**IC_{50} (µM)**	29.0	29.5

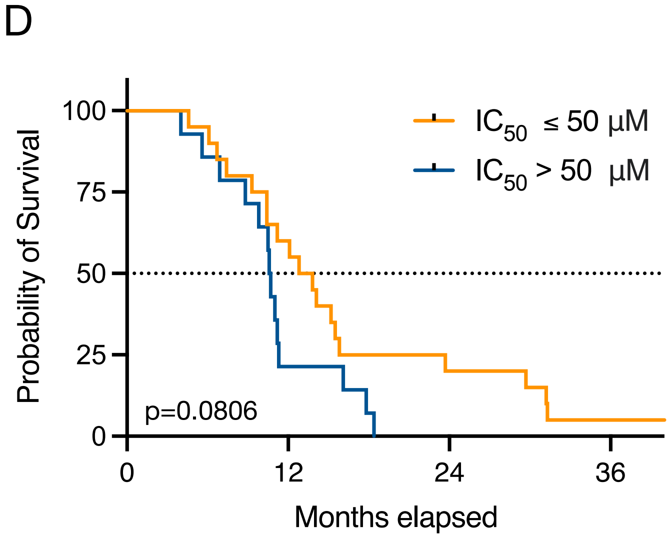

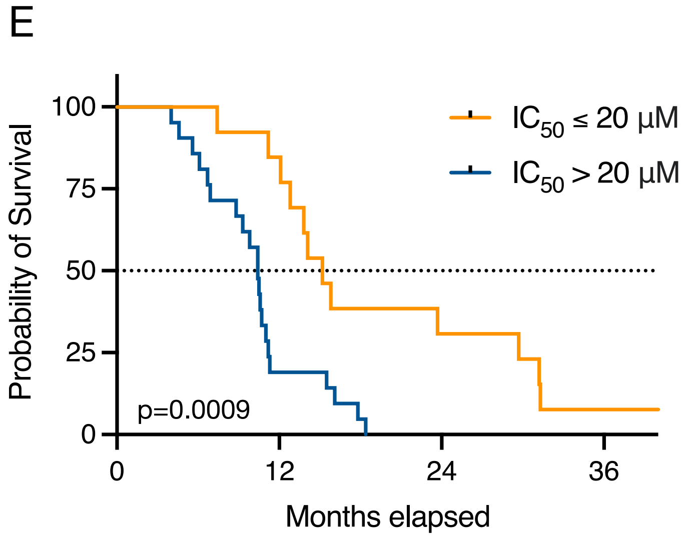

Supplementary Figure S4

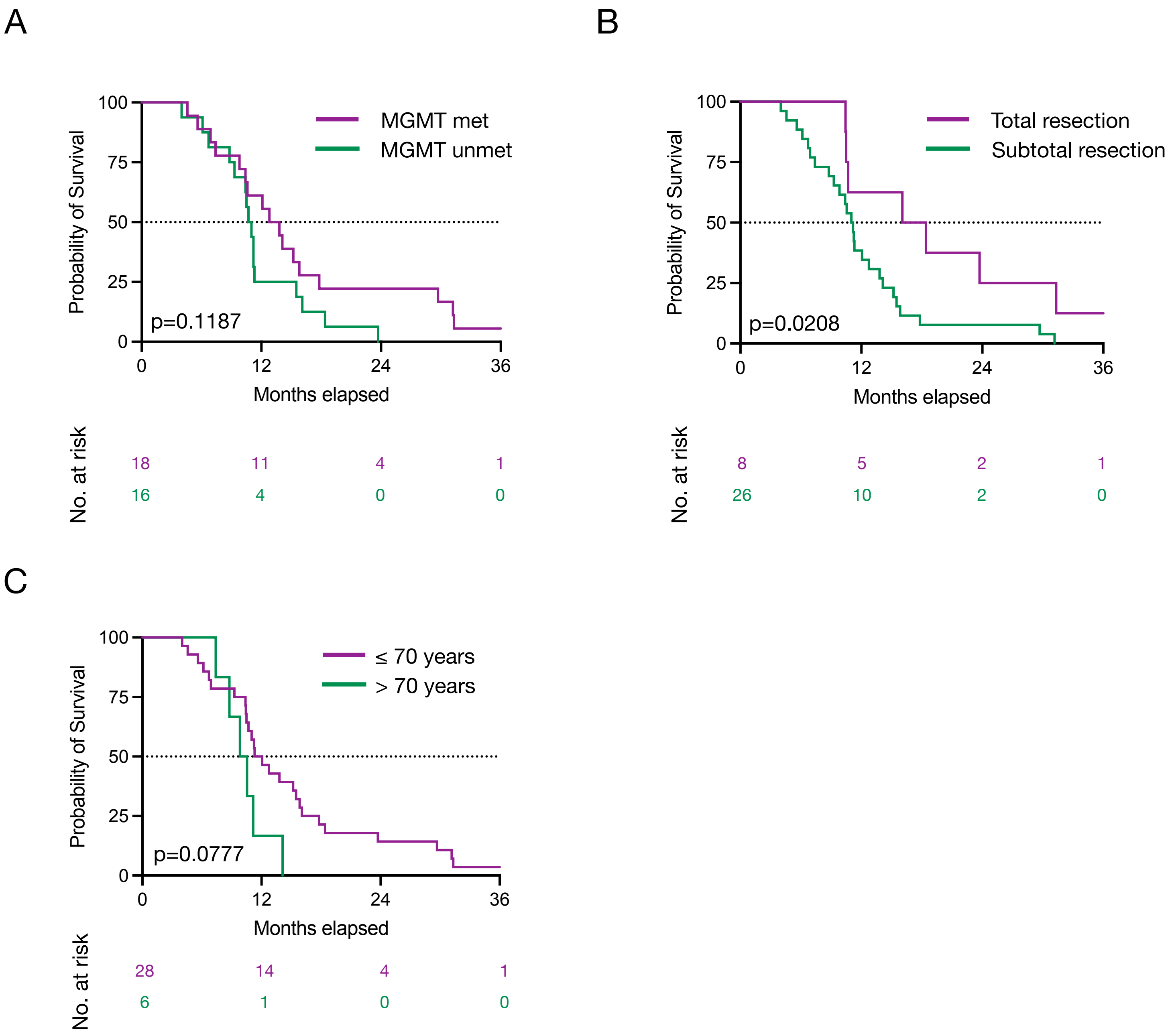

Supplement: Supplementary file 2 [file mmc2.pdf]
